# Signs of Cross‐Reactivity Between SARS‐CoV‐2 and Other Coronaviruses in a Pre‐Vaccine Swedish School Setting

**DOI:** 10.1111/apa.70532

**Published:** 2026-04-04

**Authors:** Lina Schollin Ask, Sharon Kühlmann Berenzon, Ulrika Marking, Sara Nouiser, Andreas Bråve, Sören Andersson, Charlotta Nordenhäll, Tobias Alfvén, Teghesti Tecleab, Olof Hertting, Tyler Sandberg, Wanda Christ, Jonas Klingström, Kim Blom, Charlotta Nilsson

**Affiliations:** ^1^ Unit for Vaccination Programmes, Department of Public Health Analysis and Data Management Public Health Agency of Sweden Solna Sweden; ^2^ Department of Women's and Children's Health Karolinska Institutet Stockholm Sweden; ^3^ Sachs' Children and Youth Hospital Stockholm Sweden; ^4^ Unit for Analysis, Department of Public Health Analysis and Data Management Public Health Agency of Sweden Solna Sweden; ^5^ Department of Clinical Sciences, Danderyd Hospital Karolinska Institutet Stockholm Sweden; ^6^ Department of Microbiology Public Health Agency of Sweden Solna Sweden; ^7^ School of Medical Sciences Örebro University Örebro Sweden; ^8^ Department of Global Public Health Karolinska Institutet Stockholm Sweden; ^9^ Karolinska University Hospital Stockholm Sweden; ^10^ Mabtech AB Nacka Sweden; ^11^ Division of Virology and Immunology, Department of Microbiology, Tumor, and Cell Biology Karolinska Institutet Stockholm Sweden; ^12^ Division of Molecular Medicine and Virology, Department of Biomedical and Clinical Sciences Linköping University Linköping Sweden; ^13^ Division of Clinical Microbiology, Department of Laboratory Medicine Karolinska Institutet Stockholm Sweden

**Keywords:** children, COVID‐19, immune response, SARS‐CoV‐2, school

## Abstract

**Aim:**

This pilot study explored signs of immunological cross‐reactivity between SARS‐CoV‐2 and human coronaviruses (HCoVs) in a Swedish school setting.

**Methods:**

A cross‐sectional, hypothesis‐generating study was conducted late 2020 before vaccines against COVID‐19 were available. Forty‐seven 7–8‐year‐old children, seventy‐five 14‐year‐old adolescents, fifty‐four 17‐year‐old adolescents, and 52 adult school staff were included, and immune responses to SARS‐CoV‐2 and HCoVs were assessed.

**Results:**

The SARS‐CoV‐2 seroprevalence was high in the older age groups (33%–41%). Seropositive individuals had significantly higher levels of antibodies against betacorona virus compared to seronegative individuals. SARS‐CoV‐2 seropositivity was rare among the 7–8‐year‐old children despite the high endemic context. SARS‐CoV‐2‐specific T‐cell responses in the absence of SARS‐CoV‐2‐specific antibodies were seen in all age groups.

**Conclusion:**

The data support a role for cross‐reactive immune responses induced by recent HCoV infections, with cross‐reactive T‐cell responses providing protection against COVID‐19 in young children.

## Summary

- This study investigated signs of cross‐reactive immune responses between SARS‐CoV‐2 and other human coronavirus (HCoV) in a Swedish school setting in the pre‐COVID‐19‐vaccine era.
- The data indicate cross‐reactive immune responses induced by recent HCoV infections in the youngest age groups and provide evidence for pre‐existing, cross‐reactive T‐cell immunity in children.
- The findings extend our understanding of SARS‐CoV‐2 immune responses during the early phase of the COVID‐19 pandemic.

**Abbreviations**
Ab: antibody
ANOVA: analysis of variance
AU: antibody units
BAU: binding antibody units
CPE: cytopathogenic effect
HCoV: human coronavirus
IgG: immunoglobulin G
M: membrane
MSD: Meso Scale Discovery
N: nucleocapsid
Nabs: neutralizing antibodies
PBMC: peripheral blood mononuclear cells
RBD: receptor binding domain
S: spike
S1: subunit 1 of the SARS‐CoV‐2 spike protein
S2: subunit 2 of the SARS‐CoV‐2 spike protein
SARS‐CoV‐2: severe acute respiratory syndrome coronavirus 2
SFC: spot‐forming cells

## 1 Introduction

Children and adolescents have been less prone to develop severe COVID‐19 compared to adults throughout the pandemic of SARS‐CoV‐2 [1, 2, 3], where a protective cross reactivity from recent other human coronaviruses (HCoVs) infections prior to SARS‐CoV‐2 infection has been suggested as one of several explanations [4, 5, 6, 7, 8, 9, 10, 11, 12]. Age‐dependent differences in immune responses to SARS‐CoV‐2 have been shown [13, 14, 15, 16, 17, 18]. At the time of this study, vaccines were as yet not available and primary schools were kept open for children up to the age of 16 in Sweden. In addition, there was a geographical and temporal spread of infection as well as an increasing degree of testing for SARS‐CoV‐2 [19, 20].

The aim with this study was to assess immune responses to SARS‐CoV‐2 and other HCoVs in children and staff in a Swedish school setting to explore possible cross‐reactivity.

## 2 Methods

### 2.1 Study Period and Study Population

The study was performed 16 November 2020 to 3 December 2020, when no vaccines against SARS‐CoV‐2 were yet available. It was conducted as a pilot study in a Swedish school setting in order to test logistics and laboratory methods in preparation for a study at a larger scale.

Two schools covering both primary and lower‐secondary education (1st to 9th grades) and two upper‐secondary schools (10th–12th grades) were selected by convenience within the Stockholm municipality. Children in 1st or 2nd grade (125 participants) and adolescents in 8th (127 participants) and 11th (123 participants) grade were invited, as were staff working with the students. In Sweden, most children starting 1st grade are 7 years old, while those starting 2nd grade are 8 years old, adolescents starting 8th grade are 14 years old, and those in 11th grade start at 17 years. In this study, we assumed that all children were those respective ages, while participating staff were adults at least 18 years old, in the absence of data about age. Each school was visited for 4 days by the study team, and all students and staff coming forward during those days were recruited. Written informed consent was obtained from participants either directly (staff) or via guardians (students).

### 2.2 Laboratory Analyses

Blood samples (whole blood and BD Vacutainer CPT) were collected on the schools' premises by trained nurses. The laboratory analyses were performed at the Public Health Agency of Sweden.

Total antibody (Ab) to SARS‐CoV‐2 was determined using Wantai SARS‐CoV‐2 Ab ELISA (Beijing Wantai Biological Pharmacy Enterprise Co. Ltd., Beijing, China), an assay that uses receptor binding domain (RBD) antigen and captures all antibody isotypes.

Immunoglobulin G (IgG) antibodies to SARS‐CoV‐2 full‐length spike (S), S1‐RBD, and nucleocapsid (N) and IgG antibodies to full‐length S of HCoV 229E, NL63, HKU1, and OC43 were determined using Meso Scale Discovery (MSD) V‐plex Serology Assay panel 2 and panel 3, respectively [Meso Scale Discovery Inc., Rockville, MD, USA].

A microneutralization assay was performed to screen for neutralizing antibodies (NAbs) to SARS‐CoV‐2 starting at a dilution 1:10 [21]. Results are given as either neutralizing if < 50% of the cell layer showed signs of cytopathogenic effect (CPE) or non‐neutralizing (if > 50% CPE was observed).

T‐cell responses to SARS‐CoV‐2 were assessed using the T‐SPOT *Discovery* SARS‐CoV‐2 assay kit used according to the manufacturer's instructions (Oxford Immunotech Limited, Abingdon, UK). The assay includes peptide pools covering S1, S2, N and Membrane (M). In the absence of a cut‐off given by the manufacturer, a cut‐off was set based on 213 samples' negative controls (medium only wells) and determined by the mean value +3 standard deviations giving a cut‐off of 32 spot‐forming cells (SFC)/million peripheral blood mononuclear cells (PBMCs).

### 2.3 Statistical Analyses

Analyses were conducted per age group: 7–8‐year‐old, 14‐year‐old, 17‐year‐old, and staff (adults). Results are presented as percentages, medians, and violin plots per age group.

Chi‐square tests were conducted to determine if there was evidence of differences in the proportion of seropositives among the age groups.

IgG measurements were transformed to logarithmic scale. When testing for differences among groups, we applied one‐way ANOVA when the residuals were normally distributed according to Shapiro test; if data was normal but heteroscedastic according to Levene tests, Welch's ANOVA was used; if the data was not normal, Kruskal–Wallis non‐parametric was applied. The *p*‐values were adjusted for multiple testing with Holm method. If *p*‐value from the omnibus test with more than two groups was < 0.05, we carried out *post hoc* pairwise tests with Games–Howell test when testing all groups against each other; this test adjusts for multiplicity and is valid even in the presence of heteroscedasticity without assuming normality. For IgG levels against HCoV among those seronegative for SARS‐CoV‐2, we tested each age group only against 7–8‐year‐olds and therefore applied a many‐to‐one test: Dunnett test when residuals were normally distributed, otherwise Baumgartner‐Weiß‐Schindler's non‐parametric test. The analyses were carried out in R version 4.4.0.

### 2.4 Ethical Statement

The study was approved by the Swedish Ethical Review Agency (approval number 2020‐05032, 2020‐06305, and 2021‐00613).

## 3 Results

### 3.1 Immune Responses to SARS‐CoV‐2

In total, 228 individuals from four schools in Stockholm, Sweden, agreed to participate in the study (Figure 1 and Table 1). Two of the four schools covered both primary and lower‐secondary education (1st to 9th grades), and the other two covered upper‐secondary education (10th to 12th grades). Staff were recruited from all participating schools. Serum samples from all individuals were available for antibody testing, while data for T‐cell responses was reported for 207 individuals due to incomplete sampling (4 participants) or indeterminate T‐cell results (17 participant*s*).

**FIGURE 1:**
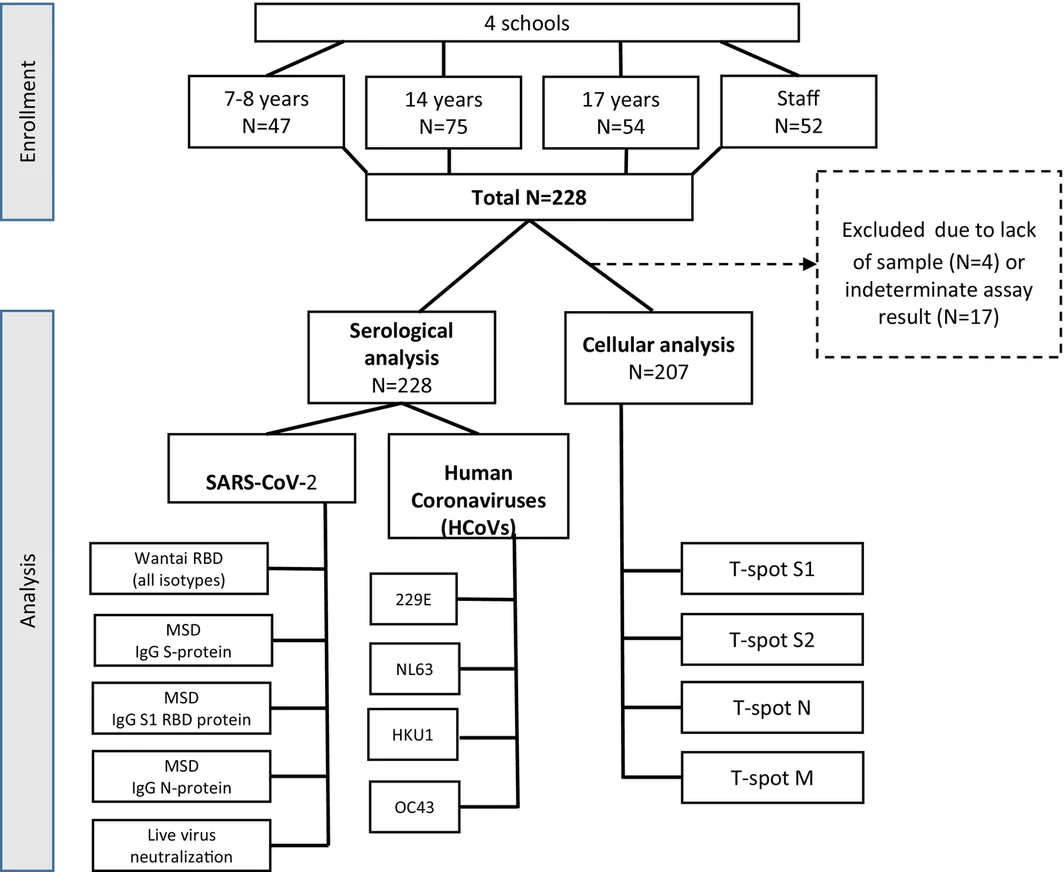
Outline of study population, study design, and the laboratory analyses performed.

**TABLE 1:** Number and proportion (%) with immune reactivity against SARS‐CoV‐2 by age group and assay. A *p*‐value for differences between age groups was obtained with chi‐square test. A *p*‐value < 0.05 was considered significant.

Assay	7–8 years *n* (%)	14 years *n* (%)	17 years *n* (%)	Staff *n* (%)	Total *n* (%)	*p*
Antibody responses	*N* = 47	*N* = 75	*N* = 54	*N* = 52	*N* = 228	
WANTAI RBD (all isotypes)	5 (11)	31 (41)	18 (33)	19 (36)	73 (32)	0.004
MSD IgG S‐protein	5 (11)	26 (35)	17 (32)	19 (36)	67 (29)	0.016
MSD IgG S1‐RBD‐protein	5 (11)	28 (37)	17 (32)	21 (40)	71 (31)	0.006
MSD IgG N‐protein	4 (8)	18 (24)	8 (15)	16 (31)	46 (20)	0.026
Live virus neutralization	5 (11)	29 (39)	16 (30)	17 (33)	67 (29)	0.01

T‐cell responses	*N* = 42	*N* = 64	*N* = 53	*N* = 48	*N* = 207	
T‐SPOT any response	9 (21)	33 (52)	20 (38)	22 (46)	84 (41)	0.004
T‐SPOT S1	7 (17)	29 (45)	16 (30)	17 (35)	69 (33)	0.021
T‐SPOT S2	8 (19)	25 (39)	16 (30)	17 (35)	66 (32)	0.168
T‐SPOT N	5 (12)	21 (33)	10 (19)	21 (44)	57 (28)	0.002
T‐SPOT M	3 (7)	13 (20)	3 (6)	4 (8)	23 (11)	0.043

Overall, antibody responses against SARS‐CoV‐2 were detected in 73 (32%) of 228 participants using the Wantai assay, while 84 (41%) of 207 participants had a T‐cell response to SARS‐CoV‐2 (Table 1).

The proportions of individuals with antibody responses to SARS‐CoV‐2 S, S1‐RBD and N as determined by MSD Mesoscale were similar between 14‐year‐old adolescents (35%, 37%, 24% respectively), 17‐year‐old adolescents (32%, 32%, 15% respectively) and staff (36%, 40%, 31% respectively), while a lower proportion of responders was seen among the group of 7–8‐year‐old children, (11%, 11%, 8%), (Table 1).

As shown in Figure 2, the magnitude of response to SARS‐CoV‐2 S, S1‐RBD and N was significantly higher among the older age groups compared to the 7–8‐year‐old children. No statistically significant difference was seen between the older age groups (all with a pairwise *p*‐value > 0.05).

**FIGURE 2:**
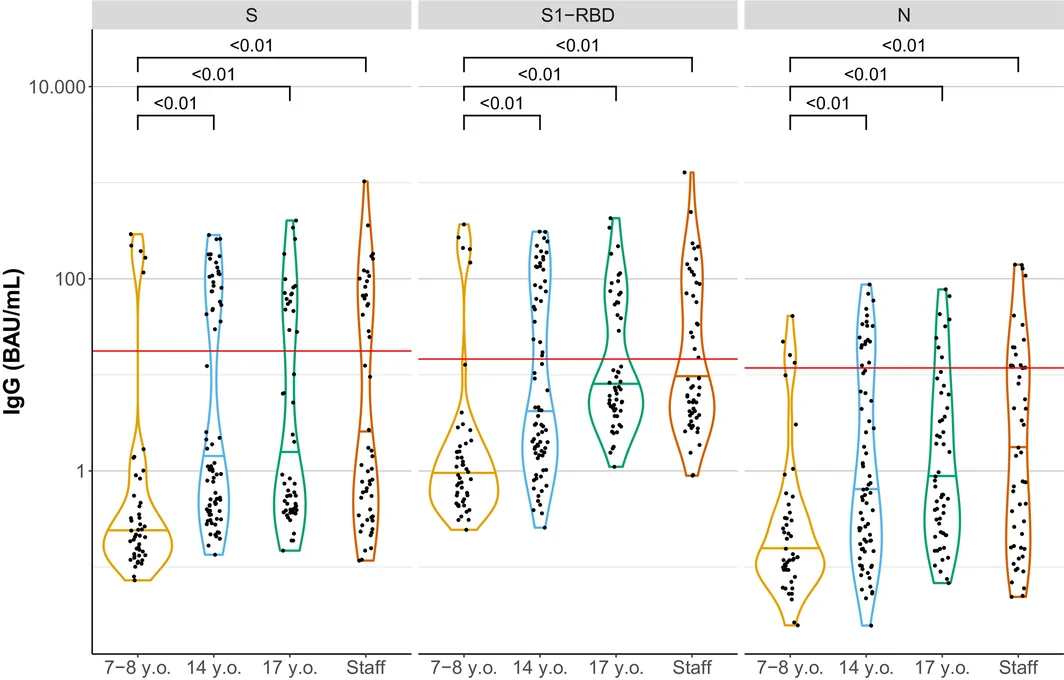
Immunglobuline G (IgG) antibodies to SARS‐CoV‐2 Spike protein (S), receptor binding domain (S1‐RBD) and Nucleocapsid protein (N) per age stratum as determined by MSD Mesoscale. Violin plots are shown as Binding Antibody Units (BAU)/ml in log scale. The cut‐off values in WHO international units are shown as horizontal red lines at 17.70 BAU/mL for S, 14.00 BAU/mL for S1‐RBD and 11.80 BAU/mL for N. Bars in the violin plots are at median values. Kruskal–Wallis omnibus test per antigen (all *p*‐values < 0.001); shown are only those *p*‐values < 0.05 from post hoc Games‐Howell pairwise‐comparison.

Neutralizing antibodies (NAb) were detected in 67 (29%) of 228 participants. The highest proportion of individuals with NAb was found among the group of 14‐year‐old adolescents (36%), followed by staff (33%) and the group of 17‐year‐old students (30%). The lowest proportion of NAb was found among the youngest age group (11%, Table 1).

A cellular (T‐cell) response to S1, S2 and N was more common among the three older age groups (45%, 39%, and 33% among 14‐year‐old adolescents, 30%, 30% and 19% among 17‐year‐olds and 35%, 35% and 44% among staff), compared to the 7–8‐year‐old children (17%, 19% and 12%). Notably, among the 14‐year‐old adolescents, 13 (20%) of 64 had an M‐specific T‐cell response, while such responses were only seen in 6%–8% of the individuals in the other age groups (Table 1 and Figure S1).

The Wantai assay that detects anti‐RBD antibodies of all immunoglobulin isotypes was used in the analyses of concurrent T‐cell‐antibody reactivity. Among the 207 individuals for which serological and T‐cell data were available, 26 (13%) had a detectable T‐cell response to SARS‐CoV‐2 in the absence of a detectable antibody response. Individuals with a T‐cell positive‐antibody negative profile were seen in all age groups, although this profile was most frequent among 14‐year‐old adolescents, (Table 2).

**TABLE 2:** Number and proportion of individuals by antibody response and T‐cell response profile to SARS‐CoV‐2 and age group.

Ab	Tc	7–8 years (%) (*N* = 42)	14 years (%) (*N* = 64)	17 years (%) (*N* = 53)	Staff (%) (*N* = 48)	Total (%) (*N* = 207)
+	+	4 (9)	21 (33)	16 (30)	17 (35)	58 (28)
−	−	32 (76)	27 (42)	31 (59)	25 (52)	115 (59)
+	−	1 (2)	4 (6)	2 (4)	1 (2)	8 (7)
−	+	5 (12)	12 (19)	4 (7)	5 (10)	26 (13)

Abbreviations: Ab, anti SARS‐CoV‐2 RBD antibodies of all subclasses; *N*, number; Tc, T‐cell reactivity against any SARS‐CoV‐2 epitope.

### 3.2 Antibody Responses to Seasonal Human Coronavirus in SARS‐CoV‐2 Seronegative Individuals

Noting that the majority of the 7–8‐year‐old children were SARS‐CoV‐2 serological non‐reactive, we probed antibody responses to HCoV 229E, NL63, HKU1, and OC43 in this group. The highest level of HCoV antibody response (median IgG level, AU/mL) in the 7–8‐year‐old SARS‐CoV‐2 seronegative children was against OC43, followed by HKU1, NL63, and 229E.

We compared HCoV antibody responses in the SARS‐CoV‐2 seronegative 7–8‐year‐old children with SARS‐CoV‐2 seronegative individuals in the other age groups. A significant difference could only be seen between seronegative 7–8‐year‐old children and seronegative 14‐year‐old adolescents when comparing 229E‐specific antibody responses (*p* = 0.011). HCoV NL63‐, HKU1‐, and OC43‐specific antibody responses were similar (*p* = 1, *p* = 0.439, and *p* = 0.797, respectively). The 17‐year‐old SARS‐CoV‐2 seronegative adolescents had significantly higher levels of HCoV 229E‐ and NL63‐specific antibodies compared to the 7–8‐year‐old children (*p* < 0.001, *p* = 0.039), but no difference was seen in HKU1‐ and OC43‐specific antibody levels (*p* = 0.929 and *p* = 0.310, respectively). The group of SARS‐CoV‐2 seronegative staff also exhibited significantly higher levels of 229E‐ and NL63‐specific antibodies (*p* < 0.001 and *p* = 0.046) compared to the youngest age group, but no difference was seen with regards to HKU1‐ and OC43‐specific antibodies (*p* = 0.27 and *p* = 0.085) (Figure 3).

**FIGURE 3:**
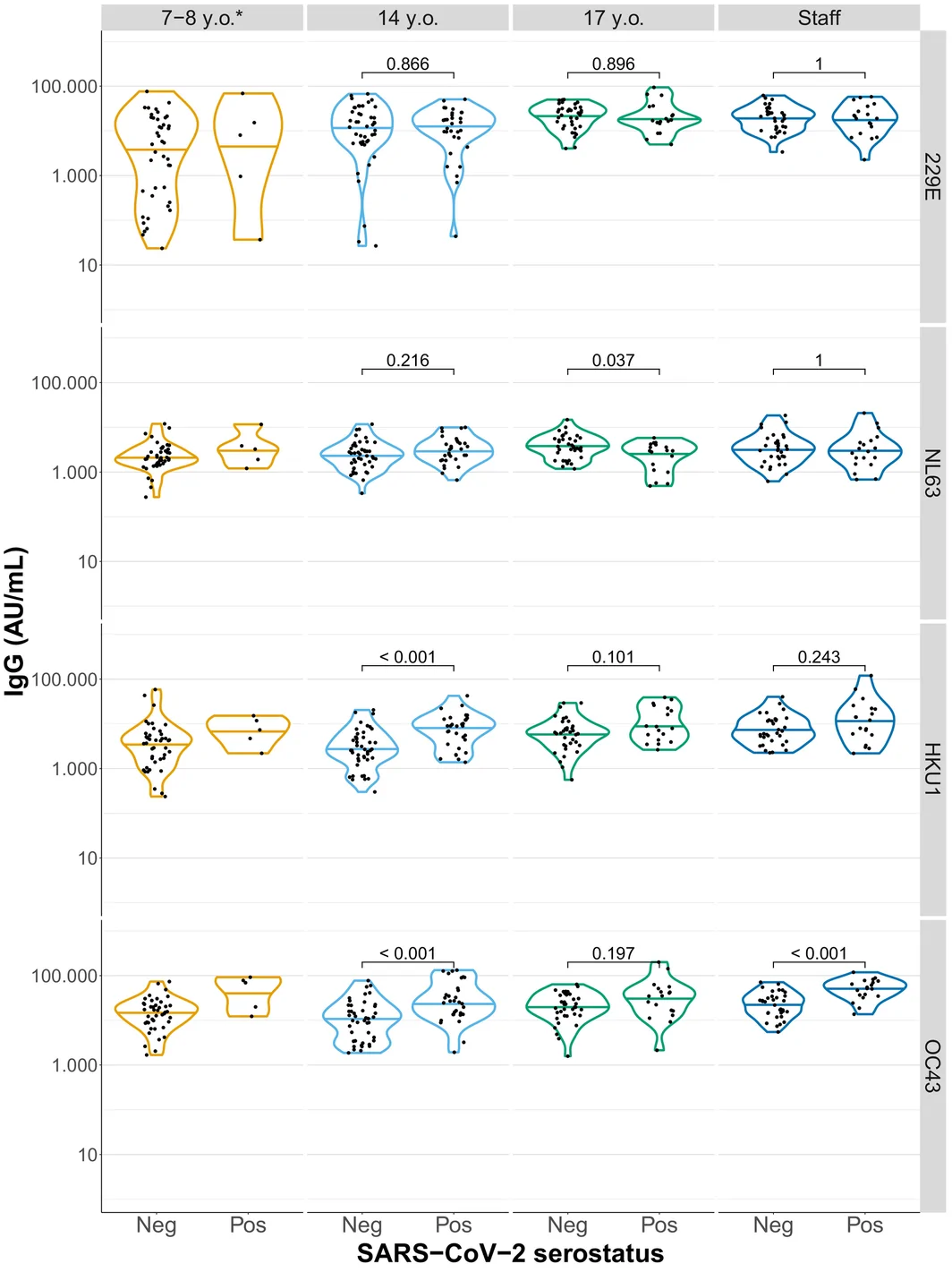
Spike IgG antibodies against human coronaviruses (HCoV) 229E, NL63, HKU1 and OC43 by SARS‐CoV‐2 serostatus (positive and negative) and per age group. Violin plots are in log AU/mL with median marked as a horizontal line. One‐way ANOVA was used to compare negative and positive SARS‐CoV‐2 serostatus in each age group and HCoV, and *p*‐values were adjusted with Holm method for multiple testing within each HCoV. The stratum 7–8 y.o. were not tested due to the small number positive for SARS‐CoV‐2 (*n* = 5). AU, antibody units.

### 3.3 Antibody Responses to Other Human β‐Corona Viruses Increased With SARS‐CoV‐2 Infection

Next, the possible interplay between immune responses to SARS‐CoV‐2 and HCoV was investigated. Individuals in three age groups (14 year‐old, 17 year‐old, and staff) were classified according to their SARS‐CoV‐2 serostatus (seropositive or seronegative by the Wantai method), and the antibody responses to HCoV 229E, NL63, HKU1, and OC43 were compared between seropositive and seronegative individuals. The youngest age group (7–8‐year‐old) was excluded from this analysis due to the low number of SARS‐CoV‐2 seropositive individuals.

As shown in Figure 3, there were significantly higher antibody responses to HCoV HKU1 and HCoV OC43 among the SARS‐CoV‐2 seropositive 14‐year‐old adolescents compared to seronegative individuals in the same age group. There were also significantly higher antibody responses to HCoV OC43 among SARS‐CoV‐2 seropositive staff compared to seronegative staff. In contrast, a SARS‐CoV‐2 seropositive status was not indicative of increased HCoV HKU‐1 antibody responses among 17‐year‐old individuals or staff, nor did the SARS‐CoV‐2 seropositive status influence HCoV OC43 among the 17‐year‐old adolescents. The SARS‐CoV‐2 seropositive status did not positively impact HCoV 229E‐ or NL63‐specific antibody responses in any age group. Among the 17‐year‐old adolescents, HCoV NL63‐specific antibody responses were lower in SARS‐CoV‐2 seropositive individuals compared to seronegative individuals.

As noted previously, we found individuals that were SARS‐CoV‐2 antibody negative in the Wantai assay and T‐cell (ELISpot) negative (Ab−/T−), SARS‐CoV‐2 antibody negative in the Wantai assay yet T‐cell positive (Ab−/T+) or SARS‐CoV‐2 antibody positive in the Wantai assay and T‐cell positive (Ab+/T+) in all study groups (7–8‐year‐old children, 14‐ and 17‐year‐old adolescents and staff, Table 2). To further understand the possible influence of HCoV responses on SARS‐CoV‐2, we compared HCoV‐specific antibodies in Ab−/T− (115 participants), Ab−/T+ (23 participants), and Ab+/T+ (58 participants) individuals. As shown in Figure 4, HCoV 229E and HCoV NL63 antibody responses were not affected by SARS‐CoV‐2 status (Ab−/T−, Ab−/T+ or Ab+/T+) (all *p*‐values > 0.05). HCoV HKU1− and HCoV OC43 antibody responses were significantly higher in Ab+/T+ than among Ab−/T− or Ab−/T+ (*p*‐values < 0.17 for all). Of note was that the Ab−/T+ group had levels of HCoV HKU1− and HCoV OC43‐specific antibodies in par with the group of non‐reactive (Ab−/T−) individuals (both *p*‐value > 0.05).

**FIGURE 4:**
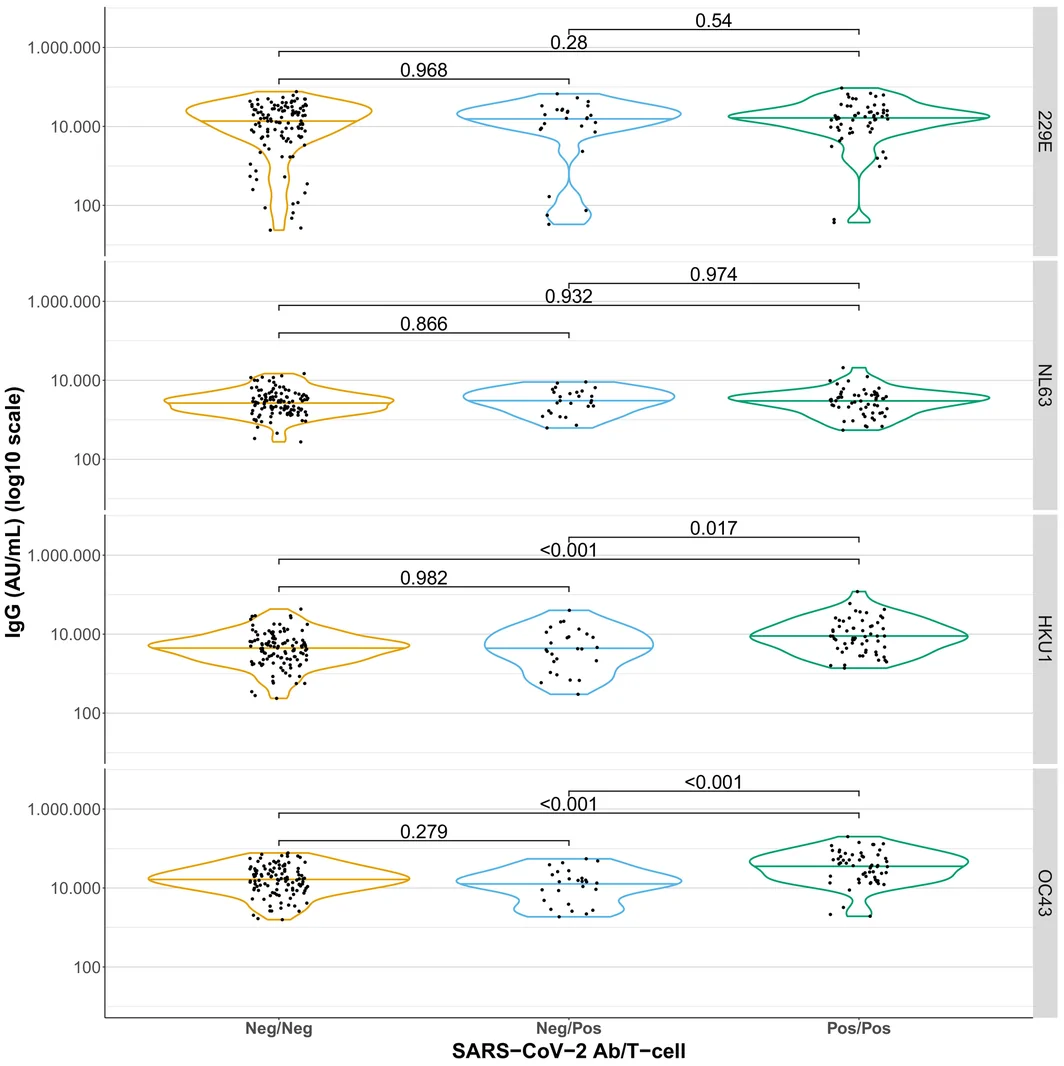
Spike IgG antibodies against human coronaviruses (HCoV) 229E, HKU1, NL63 and OC43 by SARS‐CoV‐2 reactivity profile. Neg/Neg, SARS‐CoV‐2 Wantai ELISA antibody negative and T‐cell (ELISpot) negative (Ab−/T−) (*n* = 115); Neg/Pos, SARS‐CoV‐2 Wantai ELISA antibody negative yet T‐cell (ELISpot) positive (Ab−/T+) (*n* = 26); Pos/Pos, SARS‐CoV‐2 Wantai ELISA antibody positive and T‐cell (ELISpot) positive (Ab+/T+) (*n* = 58). Violin plots are in log AU/mL with median values marked as a horizontal line. *p*‐values from post hoc Games–Howell pairwise‐comparison for each HCoV. AU, antibody units.

## 4 Discussion

We performed a cross‐sectional pilot study in a Swedish school setting to explore signs of immunological cross‐reactivity between other coronavirus infections and SARS‐CoV‐2 in the pre COVID‐19 vaccine era, November–December 2020.

We found that the prevalence of SARS‐CoV‐2 immune responses was similarly high in the study's three older age groups (14‐year‐old and 17‐year‐old adolescents and the staff), with a seroprevalence of 33%–41% and T‐cell prevalence of 38%–52%. The 7–8‐year‐old children had a comparably low SARS‐CoV‐2 seroprevalence (11%), which was intriguing considering the otherwise high‐endemic school context. Of note was that the 7–8‐year‐old children and the 14‐year‐old adolescents attended the same schools, with the 14‐year‐old adolescents having the highest SARS‐CoV‐2 seroprevalence (42%). Úbeda et al. previously reported a detectable IgG anti‐S response 3 months after exposure during lockdown 2020 in only 15 (37%) of 40 highly exposed children, and attributed protection to innate immune responses and low levels of ACE‐2 receptors [22].

Twenty‐six (13%) of 207 individuals had a SARS‐CoV‐2–specific T cell in the absence of detectable antibody responses, and this profile was seen in all four age groups, yet was most frequent among the 14‐year‐old adolescents. This profile (T cell positive yet antibody negative) has previously been reported by others [13, 23] and may suggest a recent infection with a blood draw prior to seroconversion [24] or a mild or asymptomatic infection [22]. In the present study, 20% of the 14‐year‐old adolescents had a M‐specific T‐cell response, while such T‐cell responses were rare (only seen in 6%–8%) in the other age groups. Murray et al. [23] recently reviewed experimentally defined SARS‐CoV‐2/HCoV antibody, CD4^+^ and CD8^+^ cross‐reactive epitopes. Such epitopes were frequent across several regions of the SARS‐CoV‐2 proteome, yet none were reported for the membrane protein (M), and the M‐specific T‐cell response seen here is therefore interpreted as SARS‐CoV‐2‐induced. Taken together the data suggest a shared epidemiological situation, with the 14‐year‐old adolescents having had the most recent SARS‐CoV‐2 exposure.

Pre‐existing immunity triggered by seasonal coronaviruses (HCoV 229E, NL63, HKU1, and OC43) that share cross‐reactive epitopes with SARS‐CoV‐2 has been implicated in relative clinical protection [25]. We found that SARS‐CoV‐2 seropositive 14‐year‐old adolescents and staff, but not 17‐year‐olds, had a significantly higher OC43‐specific antibody response than SARS‐CoV‐2 seronegative in the same age group. Among 14‐year‐olds, this was also shown for HKU1. This may be interpreted as a signal of cross‐reactivity between the two β‐HCoV (OC43 and HKU1) and SARS‐CoV‐2 as a result of pre‐existing antibodies or HCoV immune reactivation upon SARS‐CoV‐2 exposure [6, 7, 8, 9, 10, 13, 14, 26, 27, 28]. Dowell et al. recently compared antibody and cellular immune responses in children (aged 3–11 years) and adults and showed that antibody responses to the spike protein were high in children and that seroconversion boosted responses against β‐coronavirus (HKU1 and OC43) through recognition of the S2‐protein [13].

A study of seasonal coronavirus infections in Stockholm, Sweden between 2009 and 2020 suggested alternating peak seasons for the two α‐coronaviruses, 229E and NL63, and also for the two β‐coronaviruses, OC43 and HKU1. Due to the biennial alternating patterns, 229E and HKU1 had peak incidence in the winter seasons starting in odd‐numbered years (season 2009/2010, season 2011/2012, etc.), whereas NL63 and OC43 peaked in seasons starting in even‐numbered years (season 2010/2011, season 2012/2013, etc.) [29]. Theoretically, November 2020 would have been the start of a season of the NL63 and OC43 infections. It is notable that the highest level of antibody response in our study (performed during the 2020/2021 season) in the SARS‐CoV‐2 seronegative 7–8‐year‐old was to OC43.

Overall, the 7–8‐year‐old children in the present study exhibited a unique profile compared to the other age groups. Only five (11%) of 47 of the children in this age group were seropositive by the Wanati assay despite the high‐endemic school context. Notably, HCoV antibody responses in the SARS‐CoV‐2 seronegative 7–8‐year‐old children were as strong as those in SARS‐CoV‐2 seronegative 14‐year‐old adolescents when NL63, HKU1‐ and OC43‐specific antibody levels were compared, and as strong as those in the SARS‐CoV‐2 seronegative 17‐year‐old adolescents and staff when HKU1‐ and OC43‐specific antibody levels were compared. Moreover, 12% of the 7–8‐year‐old children exhibited a SARS‐CoV‐2 Ab−/T+ (antibody‐negative/T‐cell‐positive) profile. The strong anti‐HCoV antibody responses would support a role for pre‐existing cross‐reactive antibodies in young children. The SARS‐CoV‐2 Ab−/T+ profile could be seen as evidence for pre‐existing, cross‐reactive T‐cell responses contributing to protection against COVID‐19 in the 7–8‐year‐old children. Humbert et al. reported functional SARS‐CoV‐2 cross‐reactive CD4^+^ T‐cells in young Swedish children sampled during the pre‐pandemic era peaking at 6 years and that thereafter declined with age [15]. A recent study exploring SARS‐CoV‐2‐reactive CD4^+^ and CD8^+^ T cell function in seronegative and seropositive South African children with a median age of 7 years sampled early 2021 reported that seronegative children with CD4^+^ T cells exhibited a distinct functional profile compared to seropositive children. Furthermore, the frequency of SARS‐CoV‐2‐reactive CD4^+^ T cells was positively associated with HCoV HKU1‐spike‐specific IgG antibodies [30]. Thus, there is growing evidence for HCoV cross‐reactive T‐cell immunity playing a role in the control of SARS‐CoV‐2 in young children.

The present study was conducted prior to the access of SARS‐CoV‐2 vaccines to give insights to SARS‐CoV‐2 immune responses. It was performed as a pilot in preparation for a larger study and not designed for generalizability. Of note is that it was performed in a time period of binding regulations and recommendations during which schools for children < 16 years remained open and gatherings of > 8 were banned (starting November 8, 2020) [19].

Participants in the current study knew that they would be informed of their serostatus, which may partly explain the high overall seroprevalence (32%) as a participant bias. In comparison, a national SARS‐CoV‐2 seroprevalence study conducted 23 November to 4 December in samples from outpatients reported a seropositivity of 4.6% (95% CI 2.98–6.68) among children 0–11 years old, 5.6% (95% CI 3.31–8.69) among children 12–15 years old, 9.3% (95% CI 6.14–13.26) among youth 16–19 years old and 8.6% (95% CI 7.01–10.48) among adults 20–64 years old [20].

There are several limitations to this study. We did not collect participant demographics, and we can therefore not report data by age ranges and gender. Information about clinical COVID‐19 history and/or concurrent infections was also missing. The study was therefore based on immunological findings. Furthermore, T cell responses to HCoV and T‐cell functionality would have been useful to further decipher the interplay between SARS‐CoV‐2 and HCoV and to possibly elucidate the origin of the cross‐reactivity seen. Moreover, the four schools in Stockholm were selected by convenience and were not randomly assigned. We could therefore not control for variables such as clustering by classes and schools, which could explain some of the differences seen that may be due to lack of randomness.

In summary, the findings presented here extend our understanding of SARS‐CoV‐2 immune responses during the early phase of the COVID‐19 pandemic. The data support a role for cross‐reactive immune responses induced by recent HCoV infections in the youngest age group. The low SARS‐CoV‐2 seroprevalence in a high endemic context combined with a 12% Ab−/T+ (antibody‐negative/T‐cell‐positive) profile in the 7–8‐year‐old children could be seen as evidence for pre‐existing, cross‐reactive T‐cells providing protection against COVID‐19 in children and warrants further study.

## Author Contributions

L.S.A., S.K.B., U.M., S.N., A.B., S.A., C.No., T.A., O.H., J.K., K.B., C.Ni.: study design. L.S.A., U.M., A.B., C.No., T.A., K.B., C.Ni.: study conduct. L.S.A., S.K.B., U.M., S.N., A.B., C.No., T.A., T.T., O.H., T.S., W.C., J.K., K.B., C.Ni.: data collection and analysis. L.S.A., S.K.B., U.M., A.B., S.A., C.No., T.A., J.K., K.B., C.Ni.: Drafting the manuscript. All authors: Data interpretation and manuscript revising.

## Funding

This work was funded by a grant from the Swedish government (Ministry of Health and Social Affairs) for increased COVID‐19 testing (2020‐03‐30, S2020/02681/FS and S2020‐11‐19 S2020/08532).

## Conflicts of Interest

The authors declare no conflicts of interest.

## Supporting information


**Figure S1:** The magnitude of the T cell responses to S1, S2, N and M as determined by the T‐SPOT Discovery SARS‐CoV‐2 assay kit (Oxford Immunotech Limited, Abingdon, UK). The red line depicts the cut‐off value at 30SFC/million PBMCs. Bars in the violin plots are at median values. Values of “0” SFC/million PBMCs are arbitrarily set at 0.01 in the figure.

## Data Availability

All data generated or analysed during this study are included in this published article.
