# Supplementary material for: Signs of Cross‐Reactivity Between SARS‐CoV‐2 and Other Coronaviruses in a Pre‐Vaccine Swedish School Setting
Source: Acta Paediatr. 2026 Apr 4;115(11):2616–24. doi: 10.1111/apa.70532 (PMC13659379; doi:10.1111/apa.70532)
Supplement: Supplementary file 1 [file APA-115-2616-s001.pdf]

Signs of cross-reactivity between SARS-CoV-2 and other coronaviruses in a pre-vaccine Swedish school setting

Supplementary Material

Lina Schollin Ask[1, 2*], Sharon Kühlmann Berenzon[3], Ulrika Marking[1, 4], Sara Nouiser [5], Andreas Bråve[5], Sören Andersson[1, 6], Charlotta Nordenhäll[7], Tobias Alfvén [7, 8], Teghesti Tecleab[5], Elenor Hauzenberger[5], Olof Hertting[2, 9], Tyler Sandberg[10] , Wanda Christ[11], Jonas Klingström[5,12], #Kim Blom[5] and #Charlotta Nilsson[5,13]

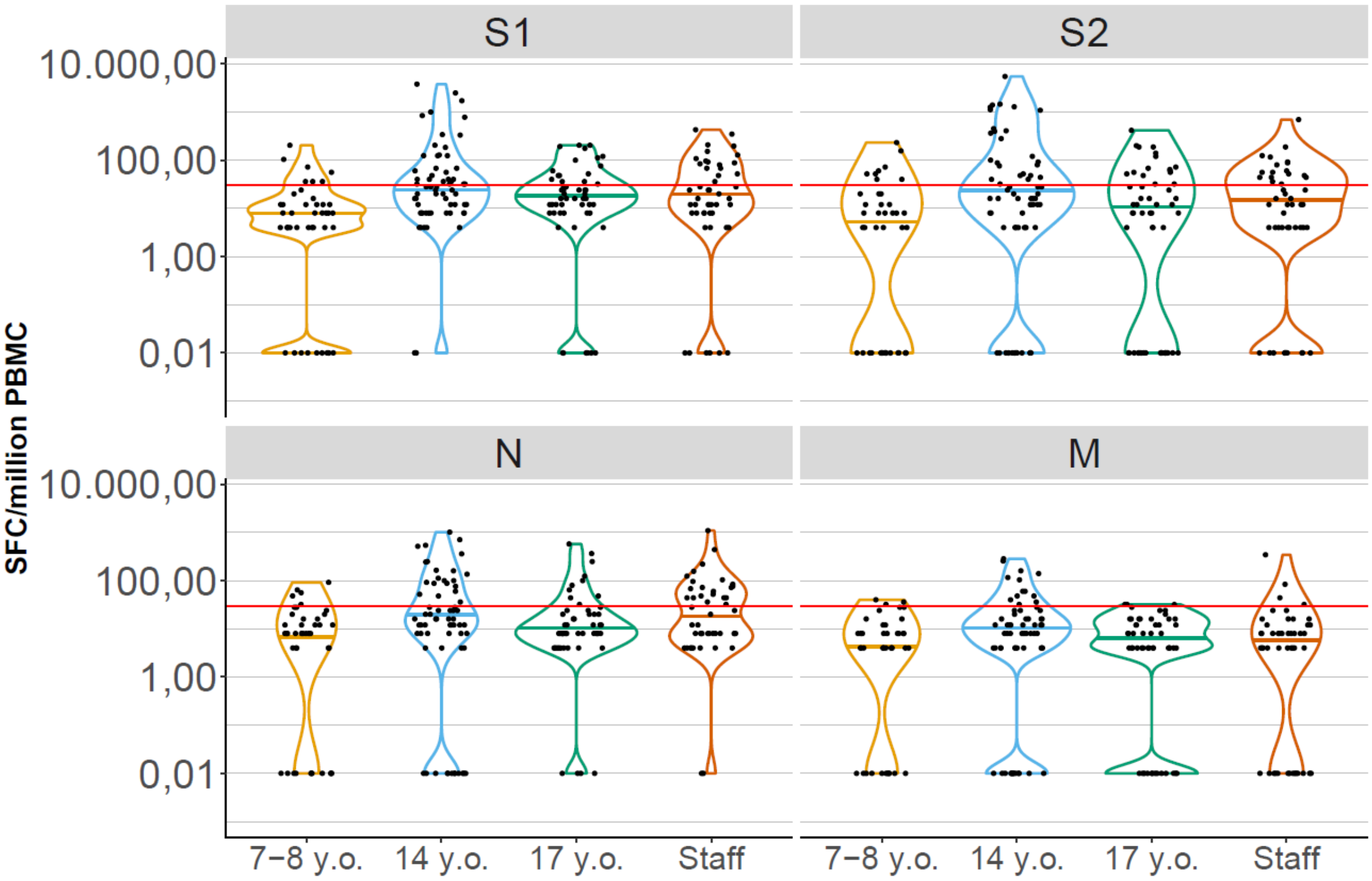

S1 Figure. The magnitude of the T cell responses to S1, S2, N and M as determined by the T-SPOT *Discovery* SARS-CoV-2 assay kit (Oxford Immunotech Limited, Abingdon, UK). The red line depicts the cut-off value at 30SFC/million PBMCs. Bars in the violin plots are at median values. Values of “0” SFC/million PBMCs are arbitrarily set at 0.01 in the figure.
